# Pembrolizumab for treatment-related neuroendocrine prostate carcinoma with a high tumor mutational burden: a case report

**DOI:** 10.3389/fonc.2025.1642412

**Published:** 2025-08-01

**Authors:** Keiji Shiba, Motohiro Fujiwara, Ayaka Onuki, Daisuke Kato, Takeshi Shirakawa, Yohei Shimizu, Takumasa Amemiya, Tsunehiro Nenohi, Yuki Matsumoto, Masayasu Urushibara, Hideto Kano, Kazuhiro Ishizaka, Mikiko Takahashi, Minato Yokoyama

**Affiliations:** ^1^ Department of Urology, Teikyo University Hospital, Mizonokuchi, Kawasaki, Kanagawa, Japan; ^2^ Department of Pathology, Teikyo University Hospital, Mizonokuchi, Kawasaki, Kanagawa, Japan

**Keywords:** pembrolizumab, prostate cancer, tumor mutational burden, treatment-related neuroendocrine prostate carcinoma, immune checkpoint inhibitors

## Abstract

Pembrolizumab has emerged as a significant therapeutic option for the treatment of solid tumors with a high tumor mutational burden (TMB-high). However, there have been no reports of its use in treatment-related neuroendocrine prostate carcinoma (t-NEPC) with TMB-high. We present the case of a 66-year-old man with metastatic prostate cancer (adenocarcinoma with a Gleason score of 4 + 5, initial prostate-specific antigen [PSA] level of 267 ng/mL, clinical stage T3bN0M1b) who was initially treated with doublet therapy, including apalutamide and leuprorelin, leading to a reduction in circulating levels of PSA < 0.001 ng/mL. However, 35 months after diagnosis, a retroperitoneal mass developed and neuron-specific enolase (NSE) levels were elevated 62.6 ng/mL. Computed tomography-guided biopsy of the tumor confirmed metastasis of t-NEPC, while genetic profiling revealed a TMB-high status. Pembrolizumab treatment was initiated at the 39-month after diagnosis. At the 41-month after diagnosis, a 75% reduction in the retroperitoneal mass and a decrease in NSE levels to 31 ng/mL were observed. This case suggests that pembrolizumab is a potential treatment option for t-NEPCs with TMB-high.

## Introduction

Prostate cancer is the most common malignancy in men worldwide, with > 1.4 million new cases reported in 2020 (1). Androgen pathway inhibition remains the standard of care for patients with metastatic prostate cancer. However, with the progression to castration-resistant prostate cancer (CRPC), the prognosis remains poor despite significant advances in treatment.

One of the major challenges in the treatment of CRPC is the development of treatment-related neuroendocrine prostate carcinoma (t-NEPC). Although most CRPC cases remain dependent on androgen receptor (AR) signaling, approximately 20% of patients eventually lose AR dependence through genetic mutations, amplification, or other mechanisms (2, 3). This leads to a histological transformation into t-NEPC, a more aggressive phenotype characterized by an AR-negative, poorly differentiated small cell morphology. The progression to t-NEPC is associated with resistance to conventional AR-targeted therapies and poor clinical outcomes (2, 4).

In recent years, the development of immune checkpoint inhibitors (ICIs) has expanded the treatment options for various cancers. ICIs enhance antitumor immunity by exposing cancer cells to immune attack. Tumors with a high burden of genetic mutations tend to be more immunogenic because of the expression of abnormal proteins known as neoantigens (5). Thus, ICI therapy has shown efficacy in patients with mismatch repair gene deficiency (dMMR). An international phase 2 trial of pembrolizumab in patients with dMMR cancers demonstrated significant efficacy regardless of tumor origin (6). Additionally, in heavily pretreated patients with unresectable or metastatic solid tumors, a high tumor mutational burden (TMB-high, defined as ≥10 mutations per megabase) was observed to be associated with an improved objective response to pembrolizumab (7). Based on these findings, pembrolizumab was approved by Food and Drug Administration (FDA) for microsatellite instability-high (MSI-H) tumors in 2017, and subsequently for TMB-high solid tumors in 2020 (7). Stemming from our daily clinical practice, we reported a case report of patients with CRPC with MSI-high who were treated with pembrolizumab administration (8).

Despite the growing interest in ICIs for prostate cancer, the occurrence of CRPC with TMB-high status remains rare, occurring in less than 5% of cases, and data on pembrolizumab efficacy in this narrowly defined patient cohort are scarce (9). Moreover, while t-NEPC is well documented, no prior case reports have described pembrolizumab treatment in a patient with t-NEPC and concurrent TMB-high status. Herein, we present the first case of a patient with TMB-high t-NEPC who was treated with pembrolizumab.

## Case presentation

A 66-year-old man with no medical history presented with lower back pain. His prostate-specific antigen (PSA) level was elevated at 267 ng/mL. The patient underwent a transperineal prostate biopsy, and the histological findings were adenocarcinoma with a Gleason score of 4 + 5. Computed tomography (CT) and bone scintigraphy demonstrated seminal vesicle invasion and spinal metastasis (Th11-S1), leading to the diagnosis of metastatic castration-sensitive prostate cancer (clinical T3bN0M1b).

**Figure 1:**
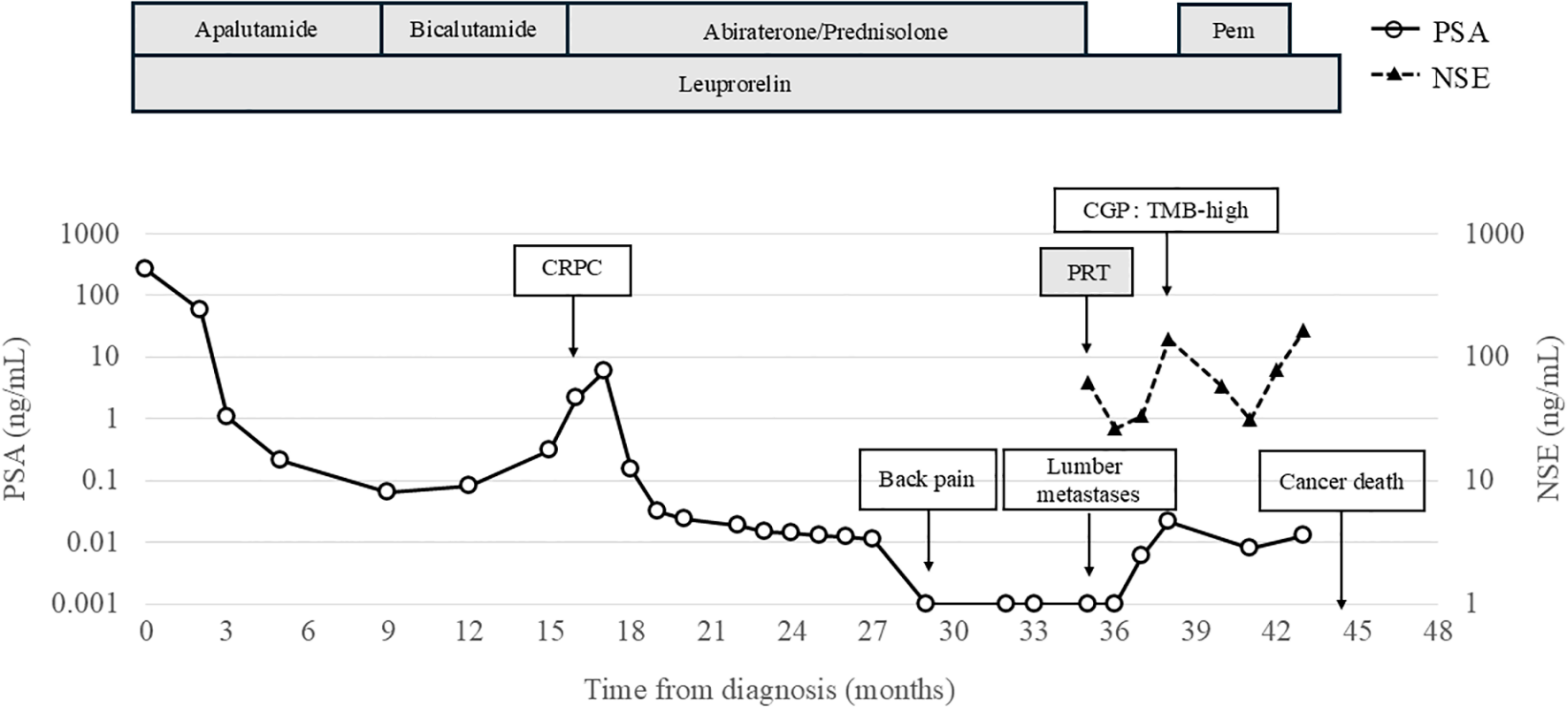
Clinical course of the case. CGP, Comprehensive Genomic Profiling; CRPC, Castration-Resistant Prostate Cancer; PSA, Prostate-specific antigen; NSE, Neuron specific enolase; Pem, Pembrolizumab; PRT, Palliative Radiotherapy.

Doublet therapy was initiated with apalutamide and leuprorelin, which resulted in a reduction of PSA levels to 0.063 ng/mL at the 9-month after diagnosis. However, owing to taste disturbances, apalutamide was discontinued and the patient was switched to bicalutamide. Subsequently, PSA levels increased to 2.162 ng/mL, and CRPC was diagnosed at the 16-month after diagnosis. A schematic representation of the clinical course of the patient is shown in **Figure 1**.

**Figure 2:**
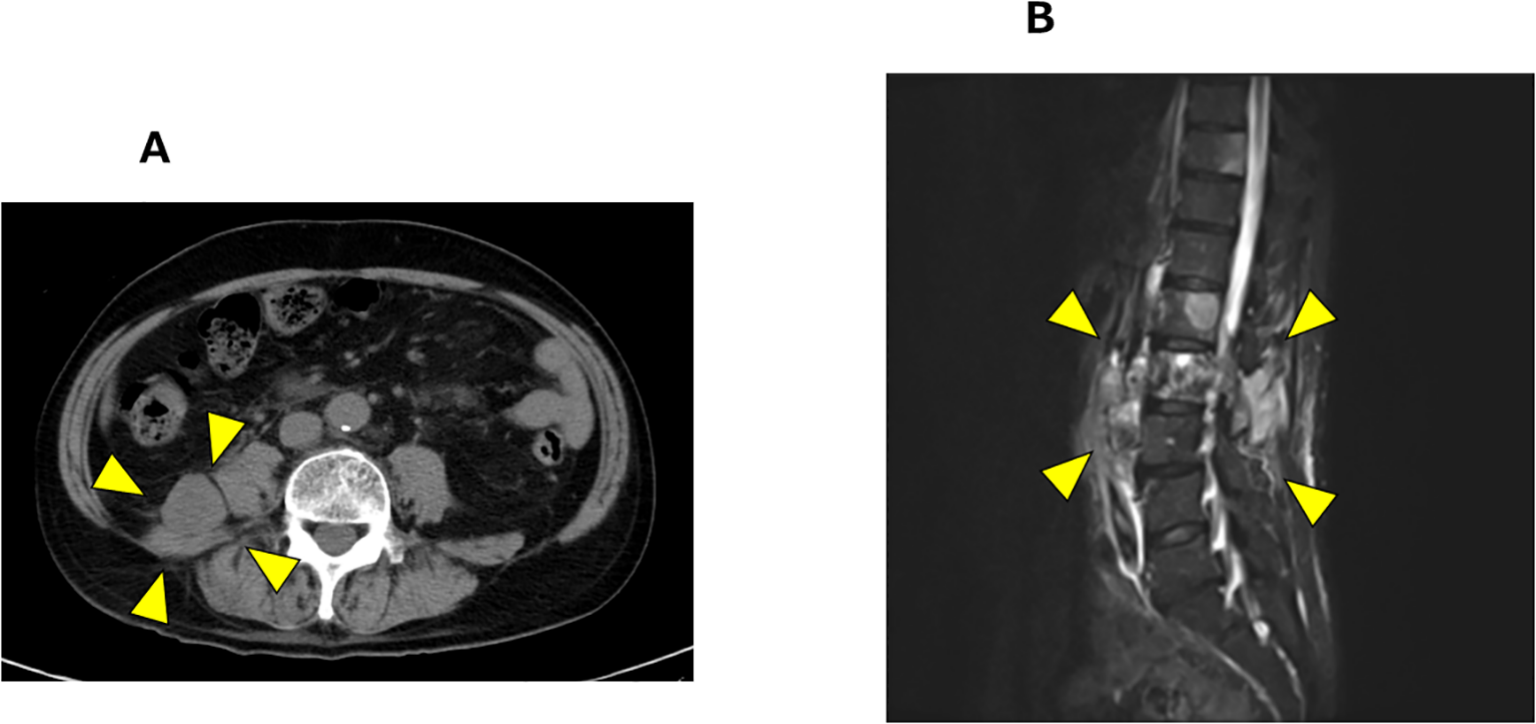
Radiological progression after abiraterone with prednisolone therapy. **(A)** Pelvic CT showing retroperitoneal mass (arrow heads). **(B)** T1 weighted MRI revealing progression of lumbar vertebral metastases (arrow heads).

**Figure 3:**
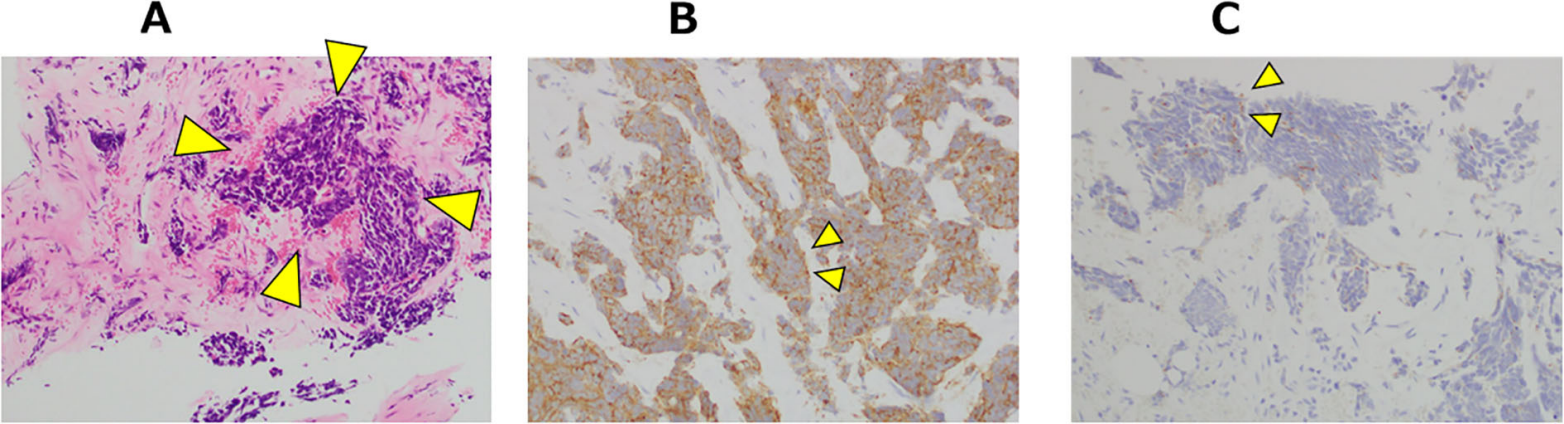
Microscopic appearance: Histological findings of the right retroperitoneal mass at the CT-guided biopsy. **(A)** Tumor cells with scant cytoplasm were distributed within the hyalinized stroma (arrow heads). Hematoxylin and eosin stain ×100. **(B)** Tumor cells were immunohistochemically positive for synaptophysin (arrow heads). ×200. **(C)** Tumor cells were immunohistochemically partial positive for chromogranin A (arrow heads). ×200.

At the 16-month after diagnosis, abiraterone therapy (1000 mg/day) with prednisolone was initiated, and the PSA levels declined to < 0.001 ng/mL. At the 29-month after diagnosis, the patient developed lower back pain; however, chest, abdominal, and pelvic CT scans revealed no new bone metastases or disease progression. At the 35-month after diagnosis, CT scans showed the presence of a retroperitoneal mass and extensive bone metastases (**Figure 2A**). Magnetic resonance imaging (MRI) revealed progression of lumbar vertebral metastases with lumbar bone marrow infiltration (**Figure 2B**). Laboratory tests revealed a PSA level of < 0.001 ng/mL and an elevated neuron-specific enolase (NSE) level of 62.6 ng/mL. Then, the patient underwent palliative radiotherapy (30 Gy/10 fractions) for the lumbar spinal metastases. At the 36-month after diagnosis, a CT-guided biopsy of the retroperitoneal mass histologically confirmed the metastasis of t-NEPC (**Figure 3**). We recommended etoposide and cisplatin therapy; however, the patient refused the treatment. At the 38-month after diagnosis, genomic profiling using FoundationOne^®^ CDx identified TMB-high status and mutations in CDK12, PTEN and STK11. No other genetic mutations, including MSI-high, were detected.

**Figure 4:**
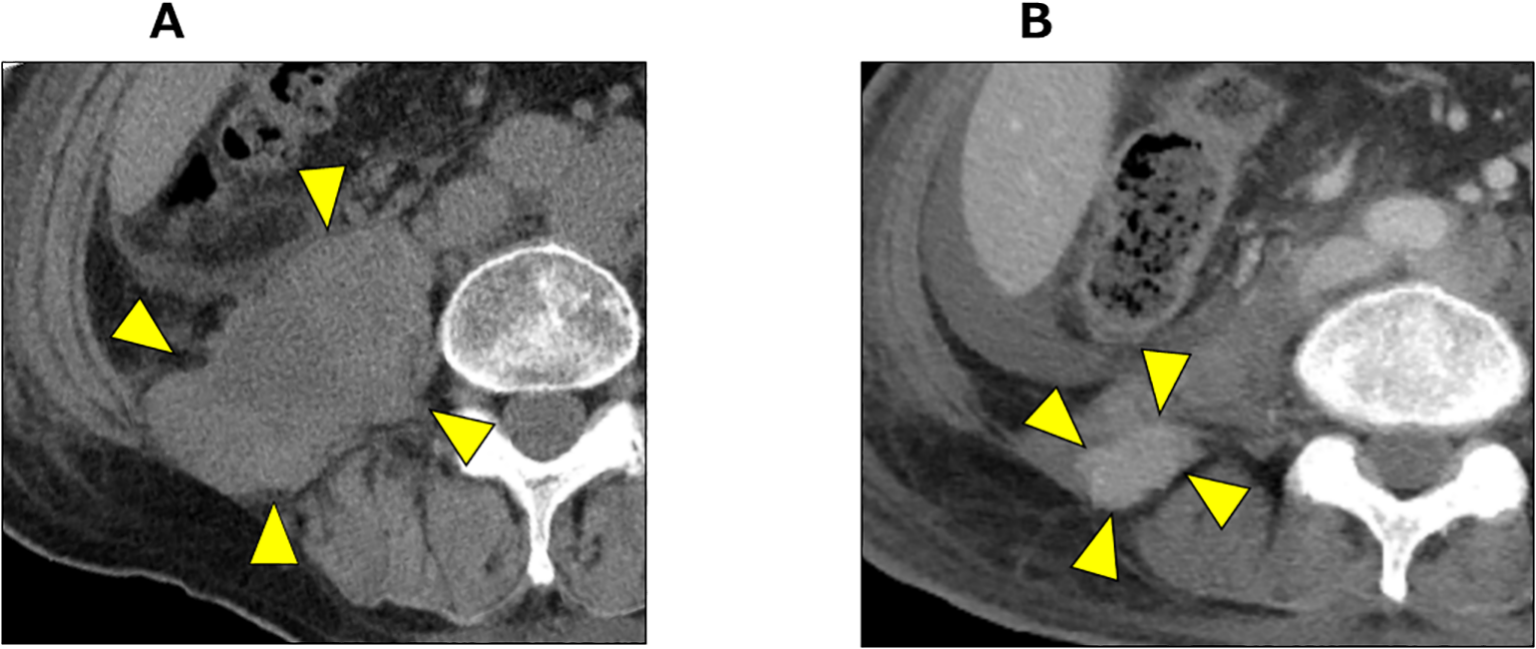
CT imaging before and after pembrolizumab administration. **(A)** CT imaging of retroperitoneal mass before pembrolizumab administration (arrow heads). **(B)** CT imaging 2 months after pembrolizumab administration. The retroperitoneal mass showed significant shrinkage (arrow heads).

At the 39-month after diagnosis, with PSA levels < 0.001 ng/mL and NSE increasing to 138 ng/mL, pembrolizumab was initiated at a dose of 200 mg every three weeks. At the 41-month after diagnosis, the NSE levels decreased to 31 ng/mL and we observed a 75% reduction (from 130 mm to 33 mm) in the retroperitoneal mass (**Figure 4**). According to Response Evaluation Criteria in Solid Tumors version 1.1 (RECIST v 1.1) criteria, this response was classified as a partial response.

At the 42-month after diagnosis, the NSE levels increased to 77.8 ng/mL, with a new pleural effusion diagnosed as malignant pleuritis by pleural fluid cytology. Pembrolizumab was discontinued at the 43-month after diagnosis due to a further increase in NSE concentration (163 ng/mL) and worsening bilateral pleural effusion. The patient passed away due to cancer progression at the 44-month after diagnosis.

## Discussion

In general, t-NEPC occurs in approximately 20% of patients with CRPC (3). The prognosis of t-NEPC cases is often poor and treatment options are limited (3, 10). In a systematic review, the prognosis of t-NEPC cases with multiple metastases was especially poor, and the median overall survival was 7 months (range, 0.5 to 63 months) (11). Conventional prostate cancer markers like PSA may not reflect disease progression in such cases, as demonstrated here (12). Marcus et al. reported that 51.8% of t-NEPC cases progress with PSA levels below 5 (13). Therefore, the follow-up of patients with CRPC should not rely solely on PSA levels, and regular imaging assessments are important.

In terms of the treatment for t-NEPC cases, no prospective studies have been conducted specifically on t-NEPC due to its rarity. Given that the combination of etoposide and cisplatin has proved effective for small cell lung cancer, this regimen has become the most widely used treatment for patients with t-NEPC due to its histological and biological similarities (14, 15). However, data on cisplatin-based chemotherapy for NEPC cases remain limited, with only a few retrospective studies available. Iwamoto et al. reported on platinum-based therapy as a first-line treatment for NEPC, with an objective response rate (ORR) of 66.7% and a median duration of response of 7.5 months (16). Recently, DeLLphi-300 study is underway evaluating the efficacy of tarlatamab as a bispecific T-cell engager (BiTE) that targets DLL3, which is highly expressed in NEPC. In the DeLLphi-300 study, ORR per RECIST v1.1 was 10.5% (95% CI, 2.9-24.8) (17). To date, no clearly defined standard treatment for t-NEPC has been established, highlighting the need for individualized therapeutic approaches (18).

Regarding TMB-high status case occurrence, the KEYNOTE-158 trial revealed that 29.3% of all neuroendocrine cancers, including prostate cancer, have a TMB-high status (19). In prostate cancer, a TMB-high status is rare in hormone-naïve prostate cancer (< 5%) and is more commonly observed in patients who have received hormone therapy for over 24 months (20). Similar to t-NEPC, no prospective studies have been conducted specifically on prostate cancer with a TMB-high status due to its rarity. The KEYNOTE-158 trial demonstrated the efficacy of pembrolizumab in treating TMB-high solid tumors, suggesting that the TMB-high status is a predictive biomarker of treatment response in a variety of cancers, including prostate cancer (7, 20, 21). The KEYNOTE-158 trial reported a higher ORR in TMB-high tumors compared to TMB-low tumors, regardless of PD-L1 expression or tumor type (21). TMB-high has many genetic mutations that allow more neo-antigens to be presented to T cells through the major histocompatibility complex (MHC) molecules. Pembrolizumab blocks programmed cell death 1 receptors and stimulates T cell activation, so the presence of TMB-high allows more neoantigens to be presented to T cells through the MHC molecules, increasing the effectiveness of pembrolizumab (5). The loss of efficacy of pembrolizumab is due to decreased expression of MHC molecules on tumor cells, as well as an increased immunosuppressive cells such as regulatory T cells (Tregs) and myeloid-derived suppressor cells. These changes impair the ability of T cells to recognize and attack tumor cells. In prostate cancer, which is often characterized as a “cold tumor” with inherently low T cells and an immunosuppressive microenvironment. Even if pembrolizumab had an initial effect, as in this case, the tumor’s immune evasion mechanism may have been further enhanced, resulting in the development of resistance to pembrolizumab (22). In the patient, this feature would have resulted in a short-term response to pembrolizumab.

In addition to the TMB-high status, an MSI-high condition has also been recognized as a biomarker for predicting the efficacy of ICIs, including pembrolizumab. Notably, 82.1% of MSI-high status tumors are also TMB-high, whereas only 18.3% of TMB-high tumors exhibit an MSI-high status (23). While pembrolizumab demonstrated an ORR of 34.3% in MSI-high status tumors, the ORR for TMB-high status tumors was 29% (7, 24). Importantly, pembrolizumab is also considered an effective treatment option for TMB-high status tumors that do not display an MSI-high condition, as in the patient described here (21). However, Mosalem et al. have reported that only one in six patients with a TMB-high status and without an MSI-high condition responded to pembrolizumab (25). Further accumulation of cases and clinical evidence are needed to clarify the therapeutic role of pembrolizumab in this patient cohort.

This report is particularly noteworthy because it described the first case of pembrolizumab treatment for a patient with CRPC with both t-NEPC and TMB-high status. The rapid tumor response observed in this case suggested that pembrolizumab may be a potential treatment for this rare subset of patients with prostate cancer. Additionally, this case highlighted the importance of genomic profiling in patients with CRPC. Although the occurrence of a TMB-high status is rare in patients with prostate cancer, its identification through genome profiling may help guide treatment decisions and enable us to use pembrolizumab in selected cases.

## Data Availability

The original contributions presented in the study are included in the article/Supplementary Material. Further inquiries can be directed to the corresponding author.
